# Supplementary material for: A study exploring procedures used to select and analyse microenterprises for persons with disabilities
Source: PLoS One. 2025 Sep 15;20(9):e0332192. doi: 10.1371/journal.pone.0332192 (PMC12435672; doi:10.1371/journal.pone.0332192)
Supplement: Attachment — Submitted filename: Response to Reviewers letter.docx [file pone.0332192.s001.docx]

**RESPONSE TO REVIEWERS (April 2025)**

We respectfully request specific, detailed, and constructive feedback to ensure clarity and transparency, allowing us to respond effectively to the team’s comments. As is, we believe that there is a lack of actionable inputs.

| **Comment from journal** | **Authors’ response** |
| --- | --- |
| **Email from Melanie Española (Straive Editorial Assistant)** | |
| If you feel you can fully address the **reviewers’** and the **Academic Editor’s comments** in a revised manuscript, you’re welcome to submit a new version of the manuscript for consideration as a new submission. | There seems to be no comments from the **reviewers**  See our response below directed to the **Academic Editor’s** comments |
| Please revise your manuscript to address the comments in the decision letter, submit your paper as a brand new submission, and do the following:  1. Refer to the previous submission (PONE-D-25-15762) in your cover letter  2. Upload a Response to Reviewers letter that includes a point by point response to each of the comments in your previous decision letter. This should be uploaded as a “Response to Reviewers” file type. | 1. Done  2. Done |
| **Academic editor:** | |
| I have prescreened the article and decided to **reject** it on the following grounds: | For a fair consideration, we respectfully request that you re-evaluate the decision, take our responses into account, and forward this manuscript to the reviewers for detailed feedback that will enable us to refine and improve the article/manuscript for publication. |
| 1. The article has not been properly written according to the PLOS One's article and coherent scientific structure. | Could you give us specifics/elaborate on this.  We followed [PLOS ONE guidelines](https://url.za.m.mimecastprotect.com/s/hEjGCKOE9MHVkZ7ouMfVc5zOgo?domain=journals.plos.org). Additionally, we reviewed PLOS ONE previous publications and referenced some, to ensure this article complies. |
| 2. Insufficient review of theory and evidence documented. | *For noting*  Available data on disability is generic. Data explicitly linked to persons with disabilities in self-employment is scarce. We observed a similar trend in a scoping review on self-employment for persons with disabilities that we are in the process of publishing. We believe this is not a prioritised research topic or area which contributes to the existing gaps.  In the same scoping review, we also found that various terms and terminologies exist to describe the same concept, indicating a lack of standardisation.  *We have added the following under:*  **Introduction**  Study contribution: Despite the content mentioned earlier, research on self-employment for individuals with disabilities remains remarkably scarce, underscoring the urgency for further investigation. Malhotra et al. (2024) (29) and Sodhi et al. (2024) (30) highlight a significant void in existing literature, emphasising the need for further exploration. Findings from a literature review on self-employment for individuals with disabilities confirm the neglect of this research area, as evidenced by the limited number of relevant articles published between 1996 and 2014 (31). This paucity of research on self-employment for persons with disabilities is further substantiated by Renko et al. (2016) (32), who state that more research on self-employment for persons with disabilities is warranted.  Especially for professionals such as occupational therapists, there is a lack of a systematic and practical approach to select and analyse suitable microenterprises for individuals with disabilities they serve. To contribute toward bridging this gap, the objectives of this study were to explore i) the selection of a suitable microenterprise for persons with disabilities and ii) factors to consider when analysing microenterprises for successful outcomes.  **The implications and recommendations:**  However, contemporary research on self-employment among individuals with disabilities continues to be lacking, necessitating further research to address this knowledge gap. As such, future studies should prioritise collecting context-relevant data and collaborating with key role players to inform evidence-based practices and policies. Thus, the recommendations from this research are summarised as follows: |
| 3. The qualitative protocol and instruments used are not clearly elaborated. | We reported in accordance to “the [Consolidated criteria for reporting qualitative research (COREQ) checklist](http://intqhc.oxfordjournals.org/content/19/6/349.long)” as suggested by PLOS ONE Submission Guidelines.  Could your team provide specific and detailed feedback on this to help us improve this manuscript. |
| 4. The qualitative analysis are not in confirmation with the key instrument used (i.e. Appendix B - Factors to consider when analysing a suitable microenterprise as a placement option). | Appendix B was removed and manuscript content revised accordingly |
| 5. Ambiguous contributions to the theory and practice in focus. | Refer to:   i. First paragraph above starting with “We respectfully request specific...” ii. Our response to Academic editor’s #2 comment above |
| Additional Editor Comments: [NOTE: If reviewer comments were submitted as an attachment file, they will be attached to this email and accessible via the submission site. Please log into your account, locate the manuscript record, and check for the action link "View Attachments". If this link does not appear, there are no attachment files.] | There are no reviewer comments or action link "View Attachments" |
