# Supplementary material for: A study exploring procedures used to select and analyse microenterprises for persons with disabilities
Source: PLoS One. 2025 Sep 15;20(9):e0332192. doi: 10.1371/journal.pone.0332192 (PMC12435672; doi:10.1371/journal.pone.0332192)
Supplement: Attachment — Submitted filename: Response_to_Reviewers_letter_auresp_1.docx [file pone.0332192.s002.docx]

**RESPONSE TO REVIEWERS (AUGUST 2025)**

**Concern:** Under “Comments to the Author”, responses between Reviewer #1 and Reviewer #2 were at times contradictory or generic, making it challenging to address such comments effectively. We enhanced the content and attempted to address all comments by revising the entire manuscript.

**Proposed way forward:** Kindly request that the reviewers provide explicit and constructive feedback, preferably directly in the manuscript. A good example of clear feedback we found helpful was provided by reviewer #2, who suggested: “Some examples from the interview questions can be added in this section.”

| **Comment from the journal** | **Authors’ response** |
| --- | --- |
| **When submitting your revision, we need you to address these additional requirements** | |
| Please ensure that your manuscript meets PLOS ONE's style requirements, including those for file naming. | Done |
| Please upload the minimal anonymized data set necessary to replicate your study findings to a stable, public repository and provide us with the relevant URLs, DOIs, or accession numbers.  Please update your Data Availability statement in the submission form accordingly | The data availability statement was updated as follows:  “All raw data files are available from Open Science Framework (OSF). The full reference is as follows: Monareng LL. PhD Raw Data. Data collection from 2023 to 2024 [Internet]. OSF; 2025. Available from: <https://osf.io/pdfks/>” |
| **Reviewers' comments: Reviewer's Responses to Questions** | |
| Is the manuscript presented in an intelligible fashion and written in standard English? PLOS ONE does not copyedit accepted manuscripts, so the language in submitted articles must be clear, correct, and unambiguous. Any typographical or grammatical errors should be corrected at revision, so please note any specific errors here. Reviewer #1: No; Reviewer #2: No | We used Grammarly (Premium) to assist with the language. This manuscript will be sent for professional language editing later, if required, after PLOS accepts it. |
| **Reviewer #1** | |
| Reviewer #1: The “Introduction” part consists of theoretical discussion. There should be a separate “Literature Review” Part. The paper should demonstrate an adequate understanding of the relevant latest literature in the field and cite an appropriate range of literature sources. | We combined the introduction with the literature for an integrated context. However, as highlighted in the manuscript, particularly in the introduction and ‘Conclusion and recommendations’, there is a paucity in the literature on self-employment for persons with disabilities in recent research.  Also, we revised the introduction section as suggested by the reviewer. The revisions include the following:  -We introduced subheadings to assist with flow and alignment.  -We revised the text to ensure alignment from the introduction to the discussion sections. |
| The “Methods” part is not well-written. The methods are not employed appropriately and able to meet the objectives of the study. | The methods, results and conclusion sections were revised. Moving forward, the reviewer should consider being more explicit, for example, by providing specific feedback in the manuscript, so we know what to amend or revise. |
| The “Results” part is not well-written. |  |
| The conclusion does not adequately tie together the other elements of the paper. |  |
| **Reviewer #2** | |
| Reviewer #2: I have listed some constructive comments to criticize the weaknesses of the paper as below. Constructive Comments: 1. At first glance, the aim, objectives, and methodology of the paper seem a bit vague. There are little adequate clarifications. I recommend to the authors to specify the aim of the study in the abstract section more appropriately. | Aim, objectives, and methodology were revised.  The aim of the study in the abstract is now clearly specified. |
| 2. In the introduction section, the authors can reflect the relevant literature review in a more systematic way. In the current form, there is a mess in the paper. In addition, the questions can be listed in the introduction to attract the attention of the readers. | Please refer to the revised and extended introduction, which is integrated with literature that aligns with the discussion. |
| 3. I recommend to the authors to clarify their methodology in detail, making sure that their planned methods/research tools are fully detailed. Some examples from the interview questions can be added in this section. The authors ought to give attention to justifying the chosen methodology in terms of demonstrating applicability, adjustment, and usefulness in the paper. | Please refer to the revised method section |
| 4. Theory-practice nexus is a good tool for a “thesis vs. counter-thesis = synthesis” approach. In this context, the way how the authors support and proof their argument and falsify their counterarguments can be more effective to keep up being focused on their story. So that the central research questions and the “argument vs. counter-arguments interactions” are streamlined in a way that addresses the research inquiries in a systematic and effective manner. Some references regarding stakeholder theory can be adapted in the theoretical framework. Thus the authors can integrate the discussion part with conclusion and recommendation section. | Refer to the revised and enhanced manuscript.  We also added a subsection under discussion titled “4.3. Implications for theory-practice integration” |
| 7. In fact, I appreciate there has been a lot of reading and ground covered, but this will not appeal to readers if the paper lacks a strong focus, compelling argument and discussion, and an indication of why the paper holds value to the readership of PLOS ONE. |  |
| 8. Finally, the investigation does not attain a theoretical saturation which comprises universally academic validity, reliability, clarity, intelligibility, and depth. Some Technical Critiques and Recommendations for the Author: 1) The author should strictly adhere to submission guidelines of the journal. 2) There are several grammatical errors and instances of badly worded/constructed sentences. Please check the manuscript and refine the language carefully. I suggest using proof reading papers before submission. | “The study adhered to the Consolidated criteria for reporting qualitative research (COREQ).” This addresses the first comment  1) Now revised  2) So far, we have used Premium Grammarly. We will engage a professional language editor later. |
